# Incidence and Long-Term Health Care Utilization Associated With Pseudomeningocele Repair Following Vestibular Schwannoma Resection: A National Database Analysis

**DOI:** 10.7759/cureus.21248

**Published:** 2022-01-14

**Authors:** Mayur Sharma, Zaid Aljuboori, Nicholas Dietz, Dengzhi Wang, Beatrice Ugiliweneza, Brian Williams, Norberto Andaluz

**Affiliations:** 1 Neurological Surgery, University of Louisville School of Medicine, Louisville, USA; 2 Neurosurgery, University of Louisville, Louisville, USA; 3 Neurosurgery, University of Louisville School of Medicine, Louisville, USA; 4 Neurosurgery, University of Louisville Hospital, Louisville, USA; 5 Kentucky Spinal Cord Injury Research Center, University of Louisville, Louisville, USA; 6 Department of Health Management and Systems Science, University of Louisville, Louisville, USA; 7 Neurosurgery, University of Cincinnati Health, Cincinnati, USA

**Keywords:** complications, neurosurgery, intracranial tumor, vestibular schwannoma, pseudomeningocele

## Abstract

Introduction

To compare the healthcare utilization in patients who presented with no pseudomeningocele (PSM) following vestibular schwannoma (VS) surgery (nd-PSM), PSM following VS surgery and required surgical repair (s-PSM) and those who presented with PSM and did not require surgical repair (ns-PSM).

Methods

MarketScan database was queried using the International Classification of Diseases, ninth and tenth revisions, and current procedural terminology four, from 2000 to 2018. We included patients *≥*18 years of age with a PSM diagnosis with at least two years of continuous enrollment. The hospital admissions, outpatient services, medication refills, and associated payments were analyzed.

Results

Of 1,460 patients, 96.6% (n=1,411) had no PSM following surgery for VS, 2.4% (n=35) were in s-PSM and only 0.95% (n=14) were in ns-PSM cohorts. Patients in the s-PSM cohort incurred higher hospital readmission rate, outpatient payments compared to those in the nd-PSM and ns-PSM cohorts at six months, one-year, and two-years following the following VS resection. At one-year following VS resection, the median combined payments for the s-PSM cohort were $74,683 compared to $42,664 for the ns-PSM and $9,476 for the nd-PSM cohort, p<0.0001. Similarly, at two-years, median combined payments for s-PSM cohort were $83,351 compared to $63,942 for ns-PSM and $18,839 for the nd-PSM cohort, p<0.0001.

Conclusion

Patients in the s-PSM cohort incurred eight times and 4.4 times the combined payments at one- and two-years, respectively, compared to the nd-PSM cohort. Also, patients in the ns-PSM cohort incurred 4.5 times and 3.4 times the payments compared to the nd-PSM cohort.

## Introduction

Cerebrospinal fluid (CSF) leak following vestibular schwannoma (VS) surgery can occur in up to 13% of patients [1-3]. Types of surgical approaches [(translabyrinthine, TL up to 13%), (middle fossa, MF and retrosigmoid, RS up to 10.6% each)] have not been shown to have an impact on the incidence of CSF leak [1,2,4]. However, other studies have reported obesity and longer operative time (>eight hours) as risk factors for CSF leak following VS surgery [3,5].

CSF leak and pseudomeningocele (PSM) following VS resection results in significant morbidity with a higher incidence of meningitis and increased utilization of health care resources [1,6]. A recent study using the American College of Surgeons National Surgical Quality (NSQIP) from 2012 to 2016 reported that CSF leak was noted in 3.7% of patients (n=37/993) and of these, 56.3% required surgical repair for CSF leak [3].

Under the current health care environment of providing value-based care and bundled payment model for surgical procedures [7-10], it is imperative to avoid complications, such as CSF leak and PSM which can significantly increase the overall health care utilization. These payment models are based on hospital performance and patient satisfaction with the idea of delivering affordable care at reasonable costs, and certainly, these complications are detrimental to the quality of life and achieving these goals. Therefore, various strategies, such as multilayered closure [11,12], using a dural substitute and resorbable mesh plate [12,13], fibrin sealant [6], using titanium mesh-hydroxyapatite cement cranioplasty [11], have been shown to mitigate the risk of CSF leak following VS resection and, therefore, health care utilization.

There is no literature regarding the long-term health care utilization of patients who developed CSF leak and PSM following VS surgery. To this end, we aimed to analyze and compare the healthcare utilization metrics in patients who presented with no PSM diagnosis following VS surgery (nd-PSM), PSM following VS surgery and required surgical repair (s-PSM), and those who presented with PSM and did not require surgical repair (ns-PSM). To the best of our knowledge, this is the first study highlighting the impact of CSF leak and PSM on health care utilization following VS surgery in patients with at least two years of follow-up.

## Materials and methods

IBM MarketScan research database was used for this study. MarketScan is a healthcare research database with de-identified medical, drug, and dental claims for more than 265 million patients. It includes inpatient, outpatient and prescription data, with diagnoses and procedures, insurer, and patient payment information [14], linked with a unique patient identification number, representing the patients’ trajectories through the healthcare system. So, it can be used to study patients’ healthcare utilization longitudinally. In our study, we used data from 2000 to 2018. The Institutional review board's approval was obtained for neurosurgical outcomes research using clinical registries and administrative and clinical databases using de-identified data; patients' consent was not required.

We analyzed the health care utilization associated with pseudomeningocele (PSM) repair following vestibular schwannoma (VS) resection. International Classification of Diseases, ninth revision (ICD-9) code 225.1 and tenth revision (ICD-10) code D33.3 were used for VS. Combined current procedural terminology (CPT) codes (61595, 61596, 61597, 61598, 61520, 61526, 61530) and (61615, 61616) were used for VS resection. Patients with both a VS diagnosis and a VS resection were selected from inpatient admission data. The first occurrence was taken as an index hospitalization.

We searched for PSM diagnosis (ICD-9 code 997.01 and ICD-10 code G97.82) and PSM surgery (CPT codes 61618 and 61619) within 90 days following VS resection, and thus, grouped our cohort into three groups: (1) no PSM diagnosis or surgery following VS resection (nd-PSM); (2) PSM surgery following VS resection (s-PSM); (3) PSM diagnosis only following VS resection and did not require surgical repair (ns-PSM).

We included adult patients 18 years and above and those who had at least two years of follow-up. The post-surgery follow-up time was calculated as the difference between the surgery discharge date and the enrollment end date (or last claim date in the dataset).

Patients’ characteristics include age, gender, insurance type (commercial, Medicaid, or Medicare) and comorbidities noted at the index hospitalization. The Elixhauser score was used to account for the burden of comorbidities [15]. We used the adaptation to ICD-9-CM codes developed by Quan et al [16].

We looked at health care outcomes including length of stay (LOS), discharge home, complication and payment during the hospital stay. We also looked at hospital readmission, outpatient services, and outpatient medication refills, associated and combined payments at six-month, one-year, and two-year follow-up. Inflation adjustments were made for payments in 2018 US dollars using the medical component of the consumer price index (accessible through the United States Bureau of Labor Statistics website) [17].

Continuous variables were summarized with means and standard deviations, median and interquartile ranges (IQRs), as well as the full range (minimum to maximum). Categorical variables were summarized with counts and percentages. Demographics and outcomes were compared using the Kruskal-Wallis test for continuous variables and the Chi-square test for categorical variables among the groups. We used the software SAS 9.4M6 (SAS Institute, Inc., Cary, NC) for data preprocessing and data analysis.

## Results

Demographics and clinical characteristics

A cohort of 1,460 patients with two years of post-VS resection follow-up was identified from the database and included in the analysis. Of 1,460 patients, 96.6% (n=1411) had no PSM following surgery for VS, 2.4% (n=35) were in s-PSM and only 0.95% (n=14) were in ns-PSM cohorts. Patients in the ns-PSM cohort were younger (median age 47 years) compared to the nd-PSM (51 years) and s-PSM (49 years) cohorts. Overall, the majority of patients were females (57%, n=836) with no difference across cohorts. Commercial insurance was the most common type of insurance used across the cohorts. The majority of patients had a comorbidity index of zero or one across the cohorts, and 21% of patients in the ns-PSM cohort had a comorbidity index of three+, Table 1.

**Table 1:** Outcomes comparisons among groups. PSM: pseudomeningocele; VS: vestibular schwanomma; nd-PSM: no PSM diagnosis following VS surgery; s-PSM: PSM following VS surgery and required surgical repair; ns-PSM: PSM following VS surgery and did not require surgical repair.

		VS resection with 2+ years follow-up				
		Total	nd-PSM	s-PSM	ns-PSM (n=14)	p-value
Demographics		(n=1,460)	(n=1,411)	(n=35)		
Age	Mean (SD)	50 (12)	50 (12)	47 (11)	46 (10)	
	Median (IQR)	51 (42, 58)	51 (42, 58)	49 (42, 57)	47 (38, 53)	0.1651
	Range (min-max)	18-82	18-82	21-62	31-61	
Gender, female, n (%)		836 (57%)	811 (57%)	18 (51%)	7 (50%)	0.6653
Insurance	Commercial, n (%)	1,269 (87%)	1,221 (87%)	35 (100%)	13 (93%)	
	Medicaid, n (%)	64 (4%)	63 (4%)	0 (0%)	1 (7%)	0.137
	Medicare, n (%)	127 (9%)	127 (9%)	0 (0%)	0 (0%)	
Elixhauser index	0, n (%)	633 (43%)	616 (44%)	14 (40%)	3 (21%)	
	1, n (%)	472 (32%)	452 (32%)	13 (37%)	7 (50%)	0.3671
	2, n (%)	226 (15%)	220 (16%)	5 (14%)	1 (7%)	
	3+, n (%)	129 (9%)	123 (9%)	3 (9%)	3 (21%)	

Health care utilization at index hospitalization, 30-days, and six months following VS resection

There was no difference in LOS, discharge to home, complications and median index payments among the cohorts at index hospitalization. However, at 30 days post-VS resection, 57% and 71% of patients in the ns-PSM cohort had ER re-admissions and complications, respectively, compared to 11% and 13% of patients in the nd-PSM cohort, p<0.0001.

At six months following index surgery, 100% of patients in the s-PSM cohort were readmitted compared to 86% of patients with PSM who were managed non-surgically and 13% of patients without PSM. Also, patients in the s-PSM and ns-PSM cohorts incurred significantly higher median payments, outpatient services, and medication refills compared to those in the nd-PSM cohort, p<0.05. The median combined payments for the s-PSM cohort were $65,743 (IQR $40,708, $95,652) compared to $32,520 (IQR $22,553, $103,991) for ns-PSM and $4,782 (IQR $1,619, $13,489) for the nd-PSM cohort, p<0.0001, Table 2.

**Table 2:** Outcomes comparisons among the groups. Bold values are significant, p<0.05. IQR: interquartile range; PSM: pseudomeningocele; VS: vestibular schwanomma; nd-PSM: no PSM diagnosis following VS surgery; s-PSM: PSM following VS surgery and required surgical repair; ns-PSM: PSM following VS surgery and did not require surgical repair.

	VS resection with 2+ years follow-up				
Outcomes	Total	nd-PSM	s-PSM	ns-PSM	p-value
	(n=1,460)	(n=1,411)	(n=35)	(n=14)	
Index hospital					
Length of hospital stay, median (IQR)	4 (3, 5)	4 (3, 5)	4 (3, 5)	5 (3, 5)	0.8315
Index payment, median (IQR)	56,045 (40,530, 85,642)	55,671 (40,490, 84,238)	68,409 (40,464, 90,908)	94,244 (46,570, 110,408)	0.145
Discharge home, n (%)	1,308 (90%)	1,264 (90%)	30 (86%)	14 (100%)	0.3345
Complications, n (%)	334 (23%)	320 (23%)	9 (26%)	5 (36%)	0.4727
Thirty-day post-discharge					
ER admission, n (%)	171 (12%)	151 (11%)	12 (34%)	8 (57%)	<0.0001>
Complications, n (%)	224 (15%)	186 (13%)	28 (80%)	10 (71%)	<0.0001>
1/2-Year post-discharge					
Hospital admissions					
Admitted, n (%)	230 (16%)	183 (13%)	35 (100%)	12 (86%)	<0.0001>
Payments, median (IQR), for admitted only	30,574 (13,824, 51,482)	24,649 (11,347, 47,307)	49,015 (32,113, 71,605)	33,090 (21,345, 114,460)	0.0004
Outpatient services					
No. of services, median (IQR)	23 (10, 47)	23 (10, 46)	44 (18, 95)	53 (29, 78)	0.0016
Payments, median (IQR)	3,546 (1,134, 8,736)	3,453 (1,122, 8,366)	9,395 (2,586, 16,679)	5,665 (1,599, 16,740)	0.0023
Medication refills					
No. of refills, median (IQR)	6 (1, 14)	6 (1, 14)	8 (4, 20)	16 (2, 24)	0.0375
Payments, median (IQR)	259 (5, 1,064)	251 (4, 1,034)	562 (96, 1,962)	338 (30, 1,718)	0.0774
Combined payments, median (IQR)	4,982 (1,683, 15,498)	4,782 (1,619, 13,489)	65,743 (40,708, 95,652)	32,520 (22,553, 103,991)	<0.0001>
One-year post-discharge					
Hospital admissions					
Admitted, n (%)	282 (19%)	235 (17%)	35 (100%)	12 (86%)	
Payments, median (IQR), for admitted only	30,097 (12,674, 56,292)	24,649 (11,281, 50,965)	49,015 (32,113, 79,976)	33,090 (21,345, 121,150)	0.0004
Outpatient services					
No. of services, median (IQR)	43 (21, 79)	42 (21, 77)	55 (29, 154)	80 (57, 134)	0.002
Payments, median (IQR)	7,121 (3,045, 15,961)	6,982 (3,019, 15,383)	16,065 (3,862, 36,978)	14,541 (3,425, 24,069)	0.0202
Medication refills					
No. of refills, median (IQR)	11 (2, 27)	11 (2, 26)	15 (5, 39)	35 (3, 48)	0.11
Payments, median (IQR)	484 (29, 2,115)	468 (27, 2,093)	1,248 (143, 3,369)	1,007 (35, 3,639)	0.2054
Combined payments, median (IQR)	9,870 (4,275, 26,290)	9,476 (4,187, 24,320)	74,683 (48,105, 120,218)	42,664 (27,766, 108,060)	<0.0001>
Two-years post-discharge					
Hospital admissions					
Admitted, n (%)	365 (25%)	318 (23%)	35 (100%)	12 (86%)	<0.0001>
Payments, median (IQR), for admitted only	29,112 (12,633, 52,495)	25,151 (11,416, 49,754)	49,045 (36,219, 93,486)	33,090 (21,345, 153,864)	<0.0001>
Outpatient services					
No. of services, median (IQR)	77 (42, 140)	77 (42, 138)	82 (55, 224)	146 (75, 265)	0.0106
Payments, median (IQR)	13,018 (6,706, 27,336)	12,817 (6,648, 26,637)	23,765 (8,720, 56,429)	18,296 (8,253, 39,996)	0.0139
Medication refills					
No. of refills, median (IQR)	22 (5, 51)	21 (5, 50)	21 (7, 78)	55 (7, 90)	0.3696
Payments, median (IQR)	1,027 (97, 4,362)	1,011 (92, 4,351)	1,906 (205, 5,155)	3,112 (74, 10,016)	0.511
Combined payments, median (IQR)	20,189 (8,941, 44,564)	18,839 (8,590, 41,424)	83,351 (50,609, 166,463)	63,942 (35,468, 112,176)	<0.0001>

Health care utilization at one and two-years following VS resection

Higher utilization of health care resources in terms of hospital readmission rate, outpatient services and corresponding higher payments were noted in patients who required surgical and conservative treatment for PSM compared to those without PSM following VS resection. Interestingly, no difference in medication refills and corresponding payments were noted among the cohorts. At one- and two-years following VS resection, median combined payments for the s-PSM cohort were $74,683 and $83,351 compared to $42,664 and $63,942 for ns-PSM and $9,476 and $18,839 for the nd-PSM cohort, respectively, p<0.0001, Table 2.

## Discussion

In this study, we found that only 3.4% of patients developed CSF leaks following VS surgery, and 71% of these patients underwent surgical repair. Patients in the s-PSM cohort incurred eight times and 4.4 times the combined payments at one- and two-years, respectively, compared to the nd-PSM cohort. Also, patients in the ns-PSM cohort incurred 4.5 times and 3.4 times the payments compared to the nd-PSM cohort at one-year and two-years following VS resection. Our study highlights the economic implications of CSF leak following VS surgery over the long term. Our results are concordant with another national database study which reported a 3.7% incidence of CSF leak following VS resection and 56.3% required surgical repair for CSF leak [3].

Posterior fossa surgeries are associated with a higher incidence of complications (up to 41%) in general and also post-operative CSF leaks [6,10,18]. Therefore, various strategies, such as meticulous multilayered closure using a dural substitute, fibrin sealant, titanium mesh, resorbable mesh cranioplasty, and cement, have been shown to reduce the incidence of CSF leak [6,11-13].

Chern et al. [12] in a recent retrospective institutional study showed that using a dural substitute and resorbable mesh plate reduced the incidence of CSF leak following the TL approach for cerebellopontine angle (CPA) tumors from 12% to 1.9%, an overall 84% reduction in CSF leak. In this study, the authors reported that the overall median cost of the procedure was $25,759.89 (range, $15,885.65-$136,433.07) using only fat grafts compared to $29,314.97 (range, $17,674.28-$111,404.55) using fat grafts + resorbable mesh. Also, the median cost of the CSF leak repair was $50,401.25 (range, $0-$384,761.71) with an additional cost of $6,048.15 in this study. However, taking into account the weighted mean cost of the resorbable mesh plat and dural substitute ($1,938.03), the overall median savings following CSF leak reduction was $3,154.51/patient [12]. In our study, we found that the overall median payment at index hospitalization for VS surgery was $56,045, compared to $55,671 for the nd-PSM cohort, $68,409 for the s-PSM cohort, and $94,244 for the ns-PSM cohort. This difference is due to the projected “cost” using institutional data in the previous study compared to the “payments” made by the insurance companies in our study. Another study showed that the CSF leak following any cranial procedures resulted in the additional cost of €1,508 ($1,773) per patient and procedure using an institutional retrospective database [6]. Additionally, the use of fibrin sealant resulted in a 63% reduction in CSF leak (10.7% to 4%), which resulted in a saving of €550 for every procedure after taking into account the cost of sealant (€400) [6].

In our study, we found that median combined payments for the s-PSM cohort were $74,683 and $83,351 compared to $42,664 and $63,942 for ns-PSM and $9,476 and $ 18,839 for the nd-PSM cohort, respectively, at one- and two-years following VS surgery. In our previous study, we reported that the combined median payments for patients who underwent stereotactic radiosurgery (SRS) for VS were $8,751 and $18,266 at one- and two-years, respectively [19]. Also, patients who underwent repeat SRS received median payments of $28,418 and $49,068 at one- and two-years, respectively. These data reflect the significant impact of a CSF leak on health care utilization in patients who underwent surgery for VS compared to SRS. Therefore, every effort should be made to avoid this complication.

Limitations

The MarketScan database is a large national administrative database that provides longitudinal analysis of a clinical problem with insights into the associated health care utilization [20]. Retrospective design, selection bias, and random sample may limit the generalizability of the results of our study. The potential for coding errors due to variation in coding for VS surgery may affect the results [21]. Also, specific patient-related data such as imaging characteristics, clinical follow-up, and type of PSM repair procedure cannot be extracted using this database. Certain complications may not be specified or reported correctly, which may account for the lower incidence of CSF leaks identified in the present analysis. The limited number of patients with the diagnosis of PSM managed conservatively may have an effect on the results. Given the small sample size, multivariable analysis was not used for statistical analysis. Despite these limitations, our study is the first one to highlight the health care utilization associated with PSM management (surgery vs. conservative) in patients who underwent surgery for VS using a large database.

## Conclusions

PSM following VS resection is a rare occurrence and the majority were managed surgically. Patients who required surgical repair for PSM (s-PSM) incurred significantly higher health care utilization followed by the ns-PSM cohort when compared to patients without CSF leak (nd-PSM cohort) at one- and two-years following VS resection. Our study provides insight regarding the impact of PSM on long-term health care utilization following VS resection. Therefore, diligent effort should be made to avoid CSF leak/PSM, which not only has a clinical impact but also on long-term health care utilization. Our study is likely to provide a baseline to compare institutional health care utilization in patients undergoing VS surgery.
